# Hydrodynamic and Thermal Slip Effect on Double-Diffusive Free Convective Boundary Layer Flow of a Nanofluid Past a Flat Vertical Plate in the Moving Free Stream

**DOI:** 10.1371/journal.pone.0054024

**Published:** 2013-03-22

**Authors:** Waqar A. Khan, Md Jashim Uddin, A. I. Md. Ismail

**Affiliations:** 1 Department of Engineering Sciences, Pakistan Navy Engineering College, National University of Science and Technology, Karachi, Pakistan; 2 School of Mathematical Sciences, Universiti Sains Malaysia, Penang, Malaysia; 3 Department of Mathematics, American International University-Bangladesh, Banani, Dhaka, Bangladesh; University of Adelaide, Australia

## Abstract

The effects of hydrodynamic and thermal slip boundary conditions on the double-diffusive free convective flow of a nanofluid along a semi-infinite flat solid vertical plate are investigated numerically. It is assumed that free stream is moving. The governing boundary layer equations are non-dimensionalized and transformed into a system of nonlinear, coupled similarity equations. The effects of the controlling parameters on the dimensionless velocity, temperature, solute and nanofluid concentration as well as on the reduced Nusselt number, reduced Sherwood number and the reduced nanoparticle Sherwood number are investigated and presented graphically. To the best of our knowledge, the effects of hydrodynamic and thermal slip boundary conditions have not been investigated yet. It is found that the reduced local Nusselt, local solute and the local nanofluid Sherwood numbers increase with hydrodynamic slip and decrease with thermal slip parameters.

## Introduction

Most physical processes (e.g. in boilers, or in a combustion engines, heat exchangers technology) involve heat generation. Normally, fluids are used to handle and transfer this heat. However, conventional heat transfer fluids (e.g. water, ethylene glycol, engine oil etc) have poor heat conductivity and require high velocities or heat transfer coefficients to efficiently transfer this heat from a given surface. To overcome these problems, Choi [1] used ultrafine nanoparticles (<100 nm in diameter) with base fluids and introduced nanofluids. Modern technologies facilitate the manufacturing of nanometer-sized particles. Various materials such as oxide ceramics (Al_2_O_3_, CuO), metal oxides (alumina, silica, zirconia, titania), carbide ceramics (SiC, TiC), chemically stable metals (gold, cupper, silver), carbon in various forms (diamond, graphite, carbon nanotubes) are often used to make ultrafine nanoparticles. Due to small sizes and very large specific surface area of the nanoparticles, nanofluids have better thermophysical properties such as high thermal conductivity, minimal clogging in flow passages, long term stability and homogeneity. Due to these improved thermophysical properties; nanofluids have diverse applications in many industries [2].

The proposed analytical model of Buongiorno [3] for convective transport in nanofluids contains Brownian diffusion and thermophoresis. This model was used by Khan and Aziz [4] to investigate the boundary layer flow of a nanofluid past a vertical surface with a constant heat flux. Kuznetsov and Nield [5] extended the classical problem of natural convection of a regular fluid over an isothermal vertical plate to the flow of a nanofluid. The gap between the work of Kuznetsov and Nield [5] and Khan and Aziz [4] has been filled by Aziz and Khan [6] by applying generalized thermal convective boundary condition to study natural convective nanofluid.

Researchers are paying their attention to investigate the double diffusive phenomena because of their many applications in chemical engineering, solid-state physics, oceanography, geophysics etc. Kuznetsov and Nield [7] studied the double-diffusive nanofluid convection in porous media. They employed Buongiorno model for the nanofluid and the Darcy model for the porous medium. They used conventional no slip boundary conditions and similarity analysis technique in their analysis. In another paper the well known Cheng-Minkowycz [8] problem was extended by Kuznetsov and Nield [9] for the double-diffusive natural convective boundary layer flow of a nanofluid in a porous medium. Recently, Khan and Aziz [10] investigated a similar problem under prescribed surface heat, solute and nanoparticle fluxes.

The above literature review reveals that all studies are restricted to conventional no slip boundary conditions. But the no-slip assumption is no longer applicable when fluid flows in MEMS and NEMS and the conventional no slip boundary conditions must be replaced by slip boundary conditions [11]. Nield and Kuznetsov [12] presented an analytic solution for forced convection flow in a parallel-plates channel or a circular duct occupied by a hyper-porous medium saturated with a rarefied gas in the slip-flow regime. The wall was subjected to uniform flux boundary conditions. They concluded that velocity slip increases heat transfer whilst the temperature slip reduces heat transfer. Kuznetsov and Nield [13] studied thermally developing forced convection in a porous medium occupied by a rarefied gas in parallel plate channel or circular tube with walls at constant heat flux. All of the above investigators applied the conventional no slip boundary conditions but there are some situations where no slip conditions lead to unrealistic behavior—for example, the spreading of a liquid on a solid substrates–, corner flow and the extrusion of polymer melts from a capillary tube (see Thompson, P.A., Troian [14]). No slip condition must be replaced by slip condition when fluid flows around microfluidic and nanofluidic (Nguyen and Wereley [15] Li [15]–[16]). The hydrodynamic and thermal slip occurs simultaneously (Karniadakis et al. [17]). The difference between the fluid velocity at the wall and the velocity of the wall itself is directly proportional to the shear stress. The proportional factor is called the slip length. The corresponding slip boundary condition is 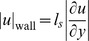, where 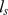 the slip length (Hak [18]). For gaseous flow the slip condition of the velocity and the jump condition of the temperature are 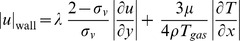 and 
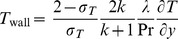
 , 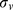 and 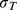 are the tangential momentum coefficient, the temperature accommodation Coefficients (Maxwell [19]). Some relevant papers on slip flows are Khare et al. [20], Petravic and Harrowella [21], Kim et al. [22], Martin and Boyd [23], Fang and Lee [24], Mathews and Hill [25], Kuznetsov and Nield [26].

The present study attempts to pinpoint the effects of the momentum and thermal slips boundary conditions on the double-diffusive free convective flow of a viscous incompressible nanofluid past a semi-infinite flat heated vertical plate in the moving free stream, which up to date have not been elucidated in the literature. In an effort to achieve these goals, we used similarity transformations to transform the governing partial differential equations into the corresponding similarity equations, before solving numerically by an implicit finite difference method. The effects of governing parameters on the similarity variables are investigated and analyzed with the help of graphical representations.

## Analysis

**Figure 1:**
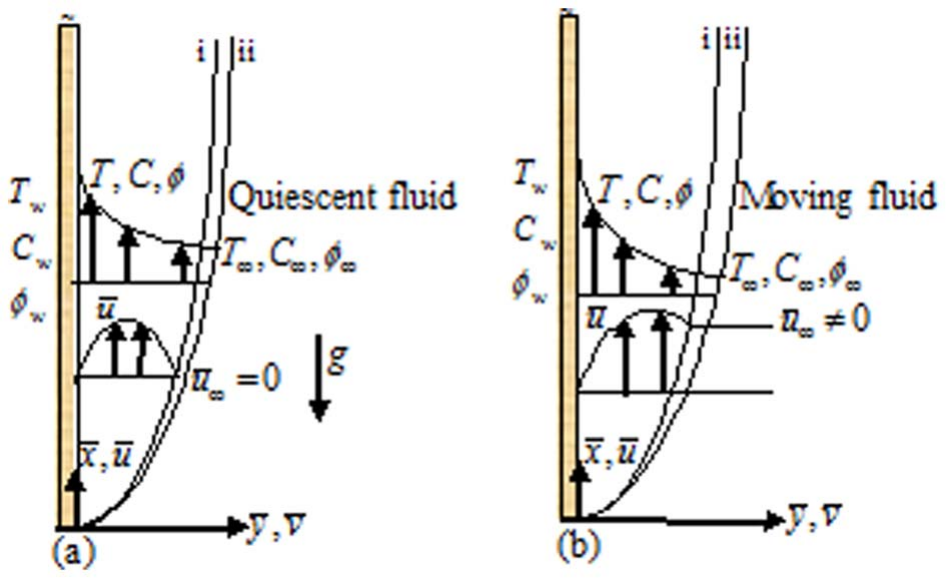
Physical model and coordinate system. (a) Fixed plate in the quiescent fluid [7]; (b) Fixed plate in the moving fluid (present).

Consider a two dimensional steady free convective boundary layer flow of water based nanofluid along a semi-infinite flat solid stationary vertical plate in the moving free stream. The coordinate system and flow configuration is depicted in **Fig. 1**. The temperature, solute concentration and nanoparticle concentration at the wall are denoted by 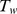 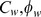. The ambient values of the temperature, solutal and nanoparticle concentration are assumed to be 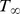, 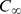 and 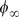. The field variables are velocity components 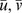, the temperature 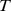, the solute concentration 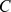 and nanoparticle concentration 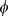. It is assumed that 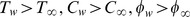 and hence a momentum, thermal, solutal and nanoparticle concentration boundary layer formed near the solid wall. In Fig. 1, i represent momentum boundary layer and ii represent thermal, solute, nanoparticle concentration boundary layers, in reality boundary layers represented by ii are not the same. We neglect viscous dissipation and Joule heating terms in the thermal equation. The Oberbeck–Boussinesq approximation is used. We include the cross diffusion terms. It is assumed that the hydrodynamic and thermal slip occur at the fluid solid interface. With these assumptions and the standard boundary layer approximations, the governing boundary layer equations in dimensional form can be written as [7].
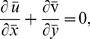
(1)

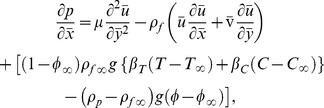
(2)

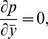
(3)

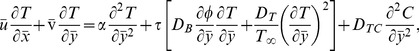
(4)

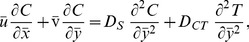
(5)

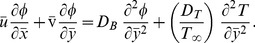
(6)


The appropriate boundary conditions are
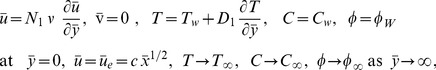
(7)


We define variables as follows: 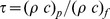: ratio of nanoparticle heat capacity and the base fluid heat capacity, 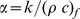: thermal diffusivity of the fluid, 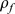: the density of the base fluid, 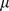,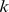: viscosity and thermal conductivity of the nanofluid, 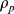: density of the particles, 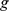: acceleration due to gravity, 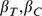: volumetric thermal expansion coefficient and volumetric solutal expansion coefficient of the nanofluid, 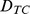: Dufour type diffusivity, 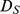: solutal diffusivity, and 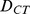: Soret type diffusivity, 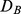: the Brownian diffusion coefficient, 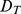: the thermophoretic diffusion coefficient, 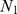: hydrodynamic slip factor with dimension (velocity)^−1^ and 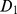: thermal slip factor with dimension length, *c* is a constant with dimension 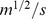 and this fact will be used in section 2.1.

### Nondimensionalization

It is suitable to express Eqs. (1)–(7) in dimensionless form, and for this purpose, we define the following dimensionless quantities:
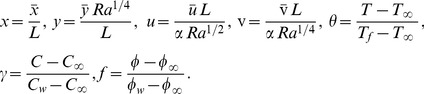
(8)Here 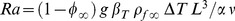 is the Rayleigh number based on the characteristic length 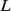. We introduce the stream function 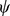 defined as 
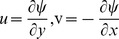
 into Eqs. (2)–(7) to reduce the number of equations and the number of dependent variables leaving the following four dimensionless equations.
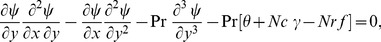
(9)

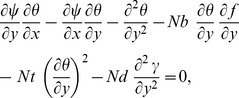
(10)

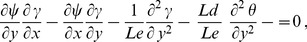
(11)

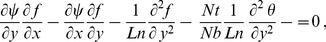
(12)subject to the boundary conditions
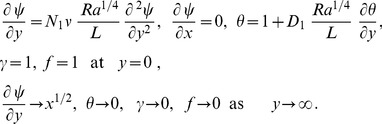
(13)


Our analysis reveals that 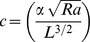.

### Similarity transformations and similarity equations

Consider the following similarity transformations developed by group method (Uddin et al. [27])
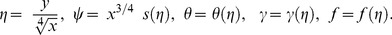
(14)In Eq. (14), 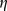 is the similarity variable, and 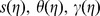 and 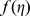 are the dimensionless stream, temperature, solutal concentration and nanoparticle concentration functions respectively.

Using Eq. (14) and Eqs. (9)–(12), leads to the similarity equations,
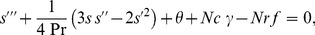
(15)

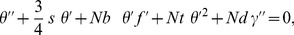
(16)

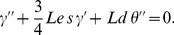
(17)

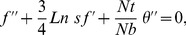
(18)subject to the boundary conditions
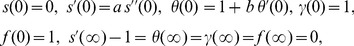
(19)Here primes denote differentiation with respect to 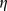 and the parameters are defined by 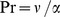 (Prandtl number), 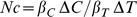 (regular double diffusive buoyancy ratio), 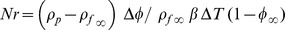 (nanofluid buoyancy ratio), 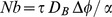 (Brownian motion parameter which is the ratio of Brownian diffusion and thermal diffusion), 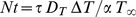 (thermophoresis parameter), 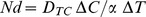 (modified Dufour parameter), parameter, 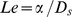 (regular Lewis number), 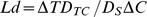 (Dufour Lewis number), 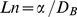 (nanofluid Lewis number), 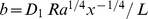
(thermal slip parameter), 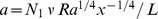 (momentum slip parameter). For true similarity solutions momentum and thermal slip parameters must be proportional to 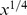. It can be noticed that for quiescent free stream, 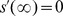, conventional no slip boundary conditions (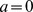) and isothermal plate (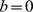) our problem reduces to that found by Kuznetsov and Nield [7].

The quantities of practical interest, in this study, are the local Nusselt number 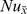, the local Sherwood number 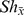 and the local nanofluid Sherwood number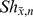, which are defined as
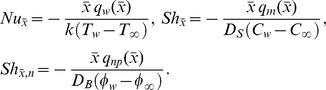
(20)


Following Kuznetsov and Nield [7], the reduced local Nusselt number 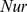, reduced local Sherwood number 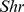 and the reduced local nanofluid Sherwood number 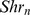 can be written as
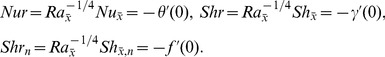
(21)


## Results and Discussion

Equations (15)–(18) subject to the boundary conditions, Eq. (19), were solved numerically using a fourth-fifth order Runge-Kutta-Fehlberg method. The step size was taken as 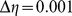 and the convergence criteria was set to 10^−6^. The asymptotic boundary conditions given by Eq. (19) were replaced by using a value of 5 for the similarity variable 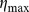 as follows.
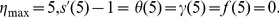
(22)


**Figure 2:**
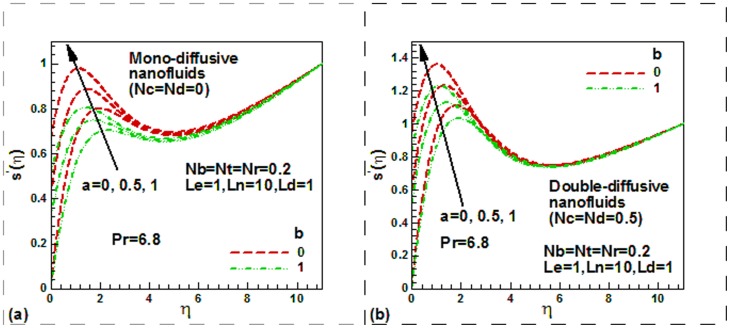
Momentum and thermal slip effect on dimensionless velocity for mono-diffusive and double diffusive water-based nanofluids.

**Figure 3:**
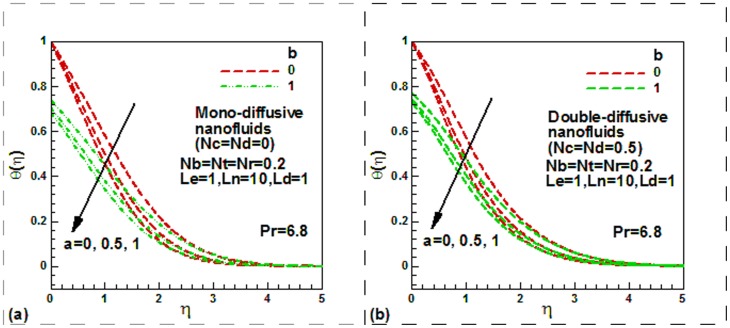
Momentum and thermal slip effect on dimensionless temperature for mono-diffusive and double diffusive water-based nanofluids.

**Figure 4:**
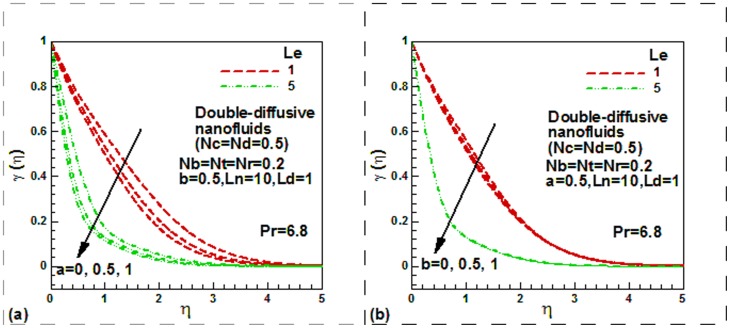
Momentum slip, thermal slip and solute Lewis number effect on dimensionless solute concentration for double diffusive water-based nanofluids.

**Figure 5:**
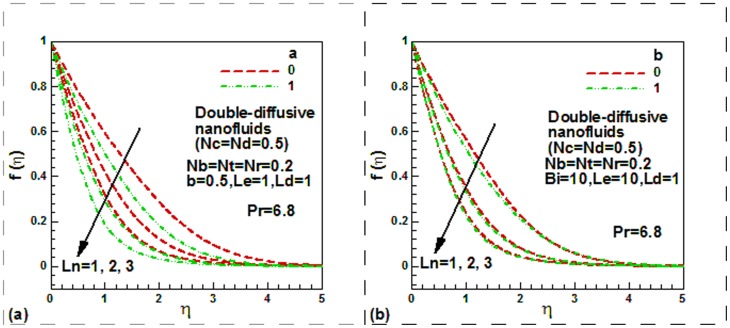
Momentum slip, thermal slip and solute nanoparticle Lewis number effect on dimensionless nanoparticles concentration for double diffusive water-based nanofluids.

**Figure 6:**
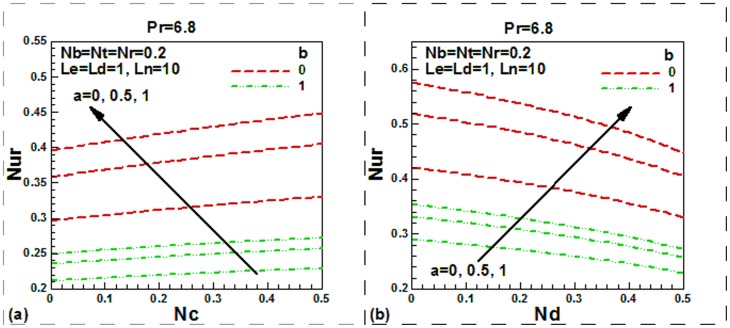
Momentum slip, thermal slip, regular double diffusive buoyancy and modified Dufour number effect on dimensionless Nesselt number.

**Figure 7:**
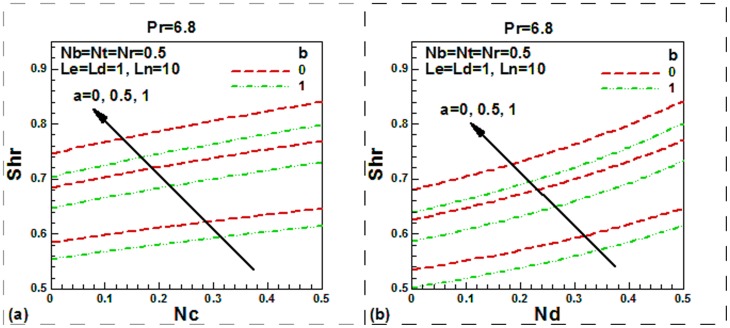
Momentum slip, thermal slip, regular double diffusive buoyancy and modified Dufour number effect on dimensionless Sherwood number.

**Figure 8:**
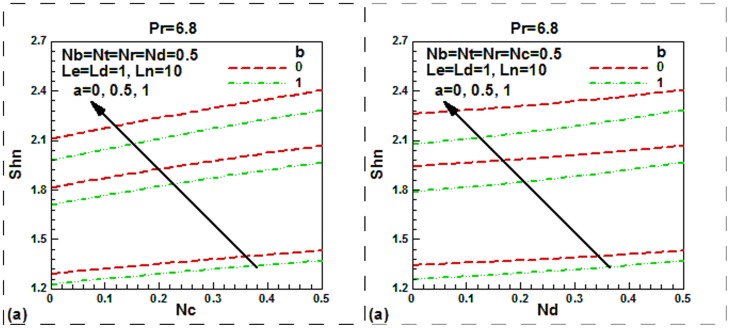
Momentum slip, thermal slip, regular double diffusive buoyancy and modified Dufour number effect on dimensionless nanoparticle Sherwood number.

The choice of 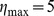 ensured that all numerical solutions approached the asymptotic values correctly. The effects of the emerging parameters on the various dimensionless functions and physical quantities are investigated and presented graphically in Figs. 2, 3, 4, 5, 6, 7, 8. The results of the reduced Nusselt number which is proportional to 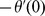 is compared for quiescent free stream, when 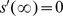, with Kuznetsov & Nield [7] for a special case which is shown in **Table 1** and found to be in good agreement. This shows the validity of our numerical results for other cases.

**Table 1:** Values of 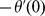 for various Pr when 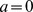 (no slip) and 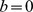 (isothermal plate).

Pr	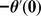	
	Quiescent free stream [7]	Quiescent free stream (present results)
0.01	0.162	0.1629
0.72	0.387	0.3909
1	0.401	0.4044
2	0.426	0.4293
10	0.465	0.4680
100	0.490	0.4909
1000	0.499	0.5010

Figure 2a shows the effect of the hydrodynamic and thermal slips on the dimensionless axial velocity for mono-diffusive for water-based nanofluids past a static plate in the moving free stream. It is apparent from Fig. 2a that the momentum slip enhances the dimensionless velocity both for the isothermal and non-isothermal plate. This is due to increase in the momentum slip which increases the velocity. From the same Fig. it is further apparent that the dimensionless velocity reduces with thermal slip for both the convectional no-slip and slip boundary conditions. It is clear that velocity is minimum for *a = 0* (no slip condition). Figure 2b shows the effect of same parameters on dimensionless velocity for double-diffusive for water-based nanofluids. It is found that dimensionless velocity enhances with the enhanced value of the momentum slip. The physical reason is exact same as explained for Fig. 2a. The effects of thermal slip on the dimensionless velocity for double-diffusive water-based nanofluids are same as mono-diffusive water based nanofluid.

Figure 3a is a plot of dimensionless temperature distribution with similarity independent variable 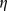 for two different values of the thermal slip parameter for mono-diffusive water based mamofluids. Note that the maximum temperature within the boundary layer reduces with rising of the momentum slip for mono-diffusive water-based nanofluids in case of either isothermal 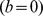 or non-isothermal 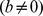 stationary plate. The maximum temperature occurs for isothermal plate. As we have seen in Fig. 2 that momentum slip increases the velocity which in turn reduces the temperature. This is what we can see from Fig. 3a. It is further seen from Fig. 3a that thermal slip causes to decrease the temperature in case of both no slip 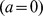 and slip 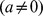 boundary condition. The physical reason is that more flow will penetrate through the thermal boundary layer due to slip effect with the increasing of 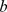. Hence more heat will be transferred and this will lead in the reduction of dimensionless surface temperature. Figure 3b exhibits the effects of thermal and momentum slip on the dimensionless temperature for double-diffusive water based nanofluids. The temperature is found to reduce with rising of the momentum slip for both isothermal 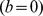 or non-isothermal 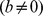 stationary plate. It is further seen from Fig. 3b that thermal slip causes to decrease the temperature in case of both no slip 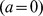 and slip 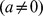 boundary condition.

The dimensionless solute concentration profiles are shown in Fig. 4(a) for mono-diffusive to show the effect of momentum slip and solutal Lewis numbers for a nonisothermal plate 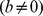. The dimensionless solute concentration decreases with an increase in momentum slip. It is clear that Lewis number reduces the dimensionless solute concentration, as expected. The physical reason is the increasing *Le* implies decreasing solute diffusivity which consequently reduced concentration and increases the mass transfer rate. Figure 4(b) is plotted to show the effect of thermal slip and solutal Lewis numbers on the dimensionless solute concentration for double-diffusive water-based nanofluids. Like momentum slip, thermal slip is found to decrease the dimensionless solute concentration. The effects of solutal Lewis numbers on solute concentration for double-diffusive nanofluid are exactly same as mono-diffusive naofluid.

The effect of momentum slip and nanoparticle Lewis number on the dimensionless nanoparticle concentration for double diffusive water-based nanofluids is displayed Fig. 5(a) whist the effect of thermal slip and nanoparticle Lewis number is shown in Fig. 5(b). Note that the dimensionless nanoparticle concentration is a decreasing function of nanoparticles Lewis number in case of both isothermal and non-isothermal plate for both mono and double diffusive water-based nanofluids. This is for both hydrodynamic slip boundary condition or for conventional no slips boundary condition.

The impact of momentum slip, thermal slip, buoyancy and modified Dufour parameters on the dimensionless reduced local Nusselt is displayed Fig. 6. The reduced local Nusselt number is increased with regular double-diffusive buoyancy ratio parameter (Fig. 6a) but it is decreased with modified Dufour parameter (Fig. 6b). In both cases, it decreases with thermal slip in case of no slip or slip boundary condition. This trends also observed by Nield and Kuznetsov [11]. It is further found that hydrodynamic slip increases the local Nusselt number both for isothermal and non-isothermal plate, as expected. A similar conclusion was also drawn by Nield and Kuznetsov [11].

Figures 7(a) and (b) aimed to shed the light on the effects of momentum slip, thermal slip, buoyancy and modified Dufour parameters on the reduced local Sherwood number. We found that the reduced local Sherwood number is an increasing function of double-diffusive buoyancy, modified Dufour and linear momentum slip parameters both for isothermal and non-isothermal plate. The reduced Sherwood number elevates in the presence of nanoparticles. This is because of the contributions of the Brownian motion, thermophoresis and the buoyant motion increased by the difference in the densities of nanoparticles and the base fluid.

Finally, the same behavior of the reduced nanofluid Sherwood number is shown in Figs. 8(a) and 8(b) for double-diffusive buoyancy and modified Dufour parameters. The reduced nanoparticle Sherwood numbers exhibited in Figs. 8(a) and 8(b) show that the reduced nanofluid Sherwood numbers is a monotonic increasing function of double-diffusive buoyancy and modified Dufour and momentum slip parameters. Observe that the Brownian motion, thermophoresis and the buoyant motion prompted by the difference in the densities of nanoparticles and the base fluid have hardly any effect on the nanoparticle Sherwood number when double-diffusion occurs.

## Conclusions

The effects of hydrodynamic and thermal slips boundary conditions on double-diffusive free convective boundary layer flow, heat and mass transfer of a nanofluid past a stationary vertical plate in moving free stream is investigated numerically. In the light of the present investigation, following conclusions can be drawn:

- The dimensionless velocity within the boundary layer increases with momentum slip but decreases with the thermal slip for both mono and double- diffusion processes in a nanofluid.
- The dimensionless temperature, solute and nanoparticle concentration decrease with both the momentum and the thermal slip.
- The dimensionless reduced local Nusselt, solute and nanofluid Sherwood numbers increase with momentum slip and decrease with thermal slip for double diffusion in nanofluids.
